# The ubiquitin ligase Nedd4-2 promotes localization of DNMBP/Tuba to P-bodies under hyperosmotic stress

**DOI:** 10.1016/j.jbc.2025.110738

**Published:** 2025-09-18

**Authors:** Zetao Liu, Chong Jiang, Faith Yeung, Brian Raught, Daniela Rotin

**Affiliations:** 1 Cell & Systems Biology Program, The Hospital for Sick Children, Toronto, Ontario, Canada; 2 Department of Biochemistry, University of Toronto, Toronto, Ontario, Canada; 3 Princess Margaret Cancer Centre, University Health Network, Toronto, Ontario, Canada; 4 Department of Medical Biophysics, University of Toronto, Toronto, Ontario, Canada

**Keywords:** NEDD4L, dynamin-binding protein, condensates, osmolarity

## Abstract

The ubiquitin ligase Nedd4-2/NEDD4L, comprised of C2-WW(x4)-HECT domains, is known to regulate several ion transporters and channels. We recently showed that elevated intracellular [Na^+^] and osmolarity enhances Nedd4-2 enzymatic activity. To globally identify its interactome and substrates in cells under hyperosmotic stress, we performed a BioID screen using miniTurbo with Nedd4-2 as a bait under hyperosmotic (vs. isosmotic) conditions. One of the top hits identified that preferentially binds Nedd4-2 under hyperosmolarity was Dynamin Binding Protein (DNMBP)/Tuba, a known GEF for Cdc42. We then showed that DNMBP is a substrate for Nedd4-2, and that active Nedd4-2 targets DNMBP to P-body condensates under hyperosmotic stress. Moreover, DNMBP itself promotes P-body formation under hyperosmolarity. Both Nedd4-2 and DNMBP are required for the activation of Cdc42 following hyperosmotic treatment, and accordingly, knockout of DNMBP results in suppression of Cdc42 and its downstream effector p38-MAPK. We thus propose that Nedd4-2–mediated targeting of DNMBP to P-bodies under hyperosmotic stress facilitates the activation of Cdc42 by this GEF.

Maintaining cellular osmolarity is essential for cell viability and growth. Elevated osmolarity in the environment (hyperosmotic stress) leads to cell shrinkage and the activation of multiple signaling pathways and cellular functions, including the activation of ion channels and transporters that act to restore normal cell volume, cytoskeleton remodeling, and gene transcription (1, 2, 3, 4). Among the cellular alterations caused by hyperosmolarity is elevated global ubiquitination levels (5, 6, 7).

Ubiquitination serves to tag proteins for degradation or for other cellular fates such as intracellular cargo trafficking, and is carried out by sequential transfer of ubiquitin from E1 to E2 and E3 enzymes, and then to substrates. The E3 ubiquitin ligase determines substrate specificity and facilitates the transfer of ubiquitin onto substrates (8, 9). However, how osmotic variations affect individual components of the ubiquitin system, especially E3 ligases, has not been investigated.

We recently found that an increase in intracellular Na^+^ concentration ([Na^+^]_i_) and elevated osmolarity enhanced the catalytic activity of Nedd4-2 (also known as NEDD4L), which is a member of HECT E3 ubiquitin ligases (10). Nedd4-2 comprises a C2-WW(x4)-HECT domain architecture, and one of its best-known substrates is the Epithelial Na^+^ channel, ENaC (11). We demonstrated that activation of ENaC, which leads to elevated [Na^+^]_i_ and osmolarity, increased Nedd4-2 activity in a process that is dependent on p38-MAPK activation and WNKs inhibition. Once activated, Nedd4-2 ubiquitinates ENaC and targets it for endocytosis and degradation (11), thus creating a negative feedback loop, whereby active ENaC promotes activation of its own suppressor, Nedd4-2 (10). However, globally, the identity and functions of proteins regulated by Nedd4-2 upon its activation by hyperosmotic stress are not known.

Here, we have identified cellular interactors of Nedd4-2 under hyperosmotic stress by performing a miniTurbo BioID screen. This screen identified Dynamin Binding Protein (DNMBP or Tuba), a specific Guanine nucleotide Exchange Factor (GEF) for Cdc42 (12), as a substrate of Nedd4-2 under hyperosmotic stress. Moreover, we show that upon hyperosmotic treatment, DNMBP localizes to P-body condensates, a process which requires the catalytic activity of Nedd4–2. Depletion/deletion of either Nedd4-2 or DNMBP leads to a reduction in Cdc42 activation under hyperosmotic stress. Finally, we show that knocking out DNMBP reduces the activation of the p38-MAPK pathway, suggesting that in hyperosmotic environment, this GEF normally promotes p38-MAPK activity *via* Cdc42 activation.

## Results

### Identification of Nedd4-2 interactors under hyperosmotic stress using BioID (miniTurbo)

**Figure 1:**
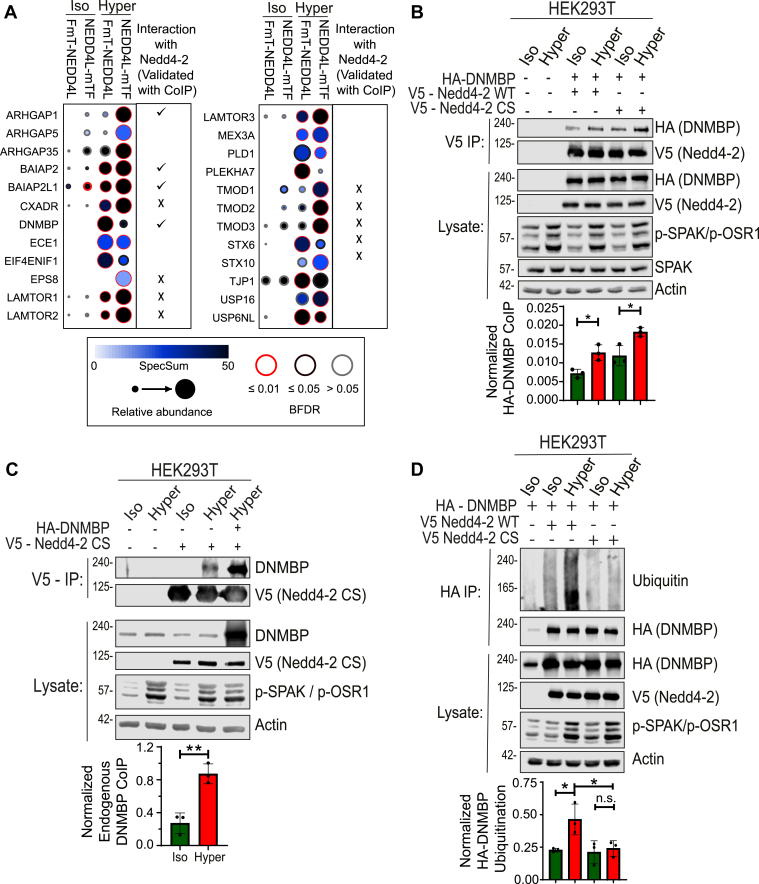
**Nedd4-2 binds and ubiquitinates Dynamin Binding Protein (DNMBP) under hyperosmotic stress**. *A*, Dot blot analysis of BioID (miniTurbo) showing *top* protein hits interacting with Nedd4-2 under hyperosmotic conditions compared to isosmotic conditions. Nedd4-2 with the miniTurbo tag (mTF) at the C-terminus is inactive. *B* and *C*, HEK293T cells were transfected (or not) with HA-tagged DNMBP and V5-tagged Nedd4-2 constructs (WT or the catalytically-inactive CS mutant) for 48 h and treated with iso- or hyper-osmotic cellular medium for 15 min Nedd4-2 was immunoprecipitated (IP) with affinity beads conjugated with anti-V5 antibodies, and HA-DNMBP or endogenous DNMBP Co-immunoprecipitation (Co-IP) was determined by immunoblotting for HA (in *B*) or DNMBP (in *C*). *p*-values were calculated using unpaired two-tailed Student’s *t* test. *D*, HEK293T cells expressing HA-DNMBP and V5-Nedd4-2 (WT or CS) were treated with iso- or hyper-osmotic solutions for 15 min. After cell lysis and lysate denaturation to eliminate non-covalently associated proteins, HA-DNMBP was immunoprecipitated with affinity beads conjugated with anti-HA antibodies, and its ubiquitination was measured by immunoblotting for Ubiquitin. *p*-values were calculated using two-way ANOVA test. Quantification is depicted below the respective blots. All data are mean ± sd, N = 3 independent experiments, *p*-values: Not significant (n.s.) > 0.05; ∗ < 0.05; ∗∗ < 0.01; ∗∗∗ < 0.001; ∗∗∗∗ < 0.0001.

We recently showed that the catalytic activity of Nedd4-2 is enhanced under hyperosmotic stress (10). To investigate the role of hyperosmolarity in regulating Nedd4-2’s function in cells, we utilized Biotin Identification (BioID) coupled with mass spectrometry to identify binding partners or potential substrates of Nedd4-2 under hyperosmotic conditions. Since ubiquitination is a rapid process, we performed BioID using the recently developed, more efficient biotin ligase, miniTurbo, which allows the identification of rapid changes in Nedd4-2 mediated interactions following acute (1 h) hyperosmotic stress. We generated two 293 Flp-In T-REx stable cell lines expressing miniTurbo-tagged Nedd4-2, with the tag placed either at the N- or C-terminus, under the control of a tetracycline-inducible promoter. N-terminal tagging preserves catalytic activity of Nedd4-2, while C-terminal tagging inhibits it (13); Active Nedd4-2 may lead to degradation/disappearance of its substrate(s), hence an inactive, C-terminal tagged Nedd4-2 was also used, to ensure trapping of potential substrates. Figure 1*A* lists several high confidence hits from the BioID screen that show enhanced binding to Nedd4-2 after hyperosmotic treatment.

### Enhanced binding of Nedd4-2 to dynamin binding protein (DNMBP) under hyperosmotic stress

To validate the interaction between Nedd4-2 and multiple proteins identified in the BioID screen, we performed co-immunoprecipitation (Co-IP) experiments (Fig. S1, *A*–*C*). One of the validated top hit proteins was Dynamin Binding Protein (DNMBP or Tuba), a GEF for the Rho GTPase Cdc42 (12). Using Co-IP, our results show that DNMBP interacts with wildtype (WT) Nedd4-2 under isosmotic conditions, and this interaction is enhanced upon hyperosmotic shock (for 15 min) in HEK293T (Fig. 1*B*) and HeLa (Fig. S1*D*) cells. Nedd4-2 binding was also observed with the endogenous DNMBP (Fig. 1*C*). (Note that hyperosmolarity was also confirmed by the increase in phosphorylation of SPAK/OSR1, downstream substrates of WNK kinases, which are well-established hyperosmotic sensors). Moreover, the interaction between DNMBP and Nedd4-2 under hyperosmolarity is further increased when cells express a catalytically inactive mutant of Nedd4-2, Nedd4-2(CS) (Figs. 1*B* and S1*D*), an effect often observed with substrates of Nedd4 proteins (14).

To further investigate whether DNMBP is a substrate of Nedd4-2, a ubiquitination assay was performed. Our results reveal a significant increase in DNMBP ubiquitination mediated by Nedd4-2 (WT) under hyperosmotic stress compared to isosmotic control. In contrast, expression of the catalytically inactive Nedd4-2(CS) mutant abolished the increase in DNMBP ubiquitination under hyperosmolarity (Fig. 1*D*). These findings suggest that DNMBP is a substrate of Nedd4-2 under hyperosmotic stress.

### Nedd4-2 interacts with the PY motifs of DNMBP

The interaction between Nedd4-2 and its substrates often occurs *via* the Nedd4-2 WW domains and the PY motif (PPxY/LPxY) of the substrate (15, 16, 17). DNMBP contains two PY motifs, LPxY, as well as a PY motif-like sequence: IPxY (Fig. S2*A*). Thus, we generated plasmids expressing DNMBP with point mutations in either one or all three PY motifs (Y145A, Y479A, Y1269A, and 3ΔPY). Subsequent Co-IP experiments were conducted to assess if Nedd4-2 interacts with DNMBP *via* its PY motifs. Our results show that under hyperosmotic stress, the interaction between DNMBP and Nedd4-2 was not affected in DNMBP containing a single point mutation in each PY motifs. However, the triple PY motif mutant of DNMBP (3ΔPY) shows a significant decrease in binding to Nedd4-2 (Fig. S2*B*). These findings demonstrate that all three PY motifs of DNMBP contribute to its binding to Nedd4-2 under hyperosmotic stress.

### Nedd4-2 and DNMBP are required to induce the activity of Cdc42 under hyperosmolarity

**Figure 2:**
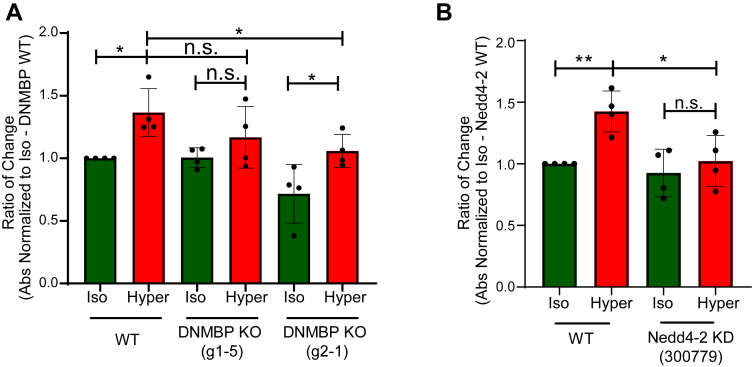
**Cdc42 activation under hyperosmolarity is reduced in DNMBP knockout (KO) and Nedd4-2 knockdown (KD) HEK293T cells.***A*, Wild-type (WT) or DNMBP KO HEK293T cells (Clone g1-5 and g2-1) were treated with iso- or hyper-osmotic cellular medium for 15 min Cdc42 activity was measured using the G-LISA activation assay. *p*-values were calculated using two-way ANOVA test with Tukey’s multiple comparison test. *B*, WT or Nedd4-2 KD HEK293T (shRNA: 300,779) cells were incubated in iso- or hyper-osmotic medium for 15 min Cdc42 activity was measured using the G-LISA activation assay. *p*-values were calculated using a two-way ANOVA test with Sidak’s multiple comparison test. All data are mean ± sd, N = 4 independent experiments, *p*-values: Not significant (n.s.) > 0.05; ∗ < 0.05; ∗∗ < 0.01.

It was previously shown that Cdc42 activity is increased under hyperosmotic conditions (18). To investigate if DNMBP is the GEF that exchanges the GDP to GTP on Cdc42 after hyperosmotic shock, we generated monoclonal DNMBP CRISPR Knockout (KO) HEK293T stable cell lines (Fig. S3*A*). The activity of Cdc42 was then measured using an ELISA-based assay in WT or DNMBP KO cells under iso- or hyper-osmotic conditions. Our results show that knocking out endogenous DNMBP suppresses Cdc42 activation after hyperosmotic treatment (Fig. 2*A*), although we observed some variability in this suppression between different DNMBP-KO clones. Since Cdc42 regulates actin cytoskeleton organization (3), we analyzed the effect of DNMBP knockout on the actin cytoskeleton. Our results reveal partially disrupted actin filaments in the DNMBP-KO cells under hyperosmolarity, consistent with reduced Cdc42 activity (Fig. S3*B*).

Furthermore, monoclonal Nedd4-2 knockdown (KD) in HEK293T cells (Fig. S3*C*) was used to test if Nedd4-2 is required for the enhanced activation of Cdc42 under hyperosmolarity. Our work shows that depleting Nedd4-2 abolishes the enhanced activation of Cdc42 upon hyperosmotic shock (Fig. 2*B*). Overall, these results indicate that DNMBP is a GEF that stimulates activation of Cdc42 under hyperosmolarity, an effect that requires Nedd4-2.

### Hyperosmolarity induces DNMBP accumulation in puncta in cells, a process that requires Nedd4-2 activity

**Figure 3:**
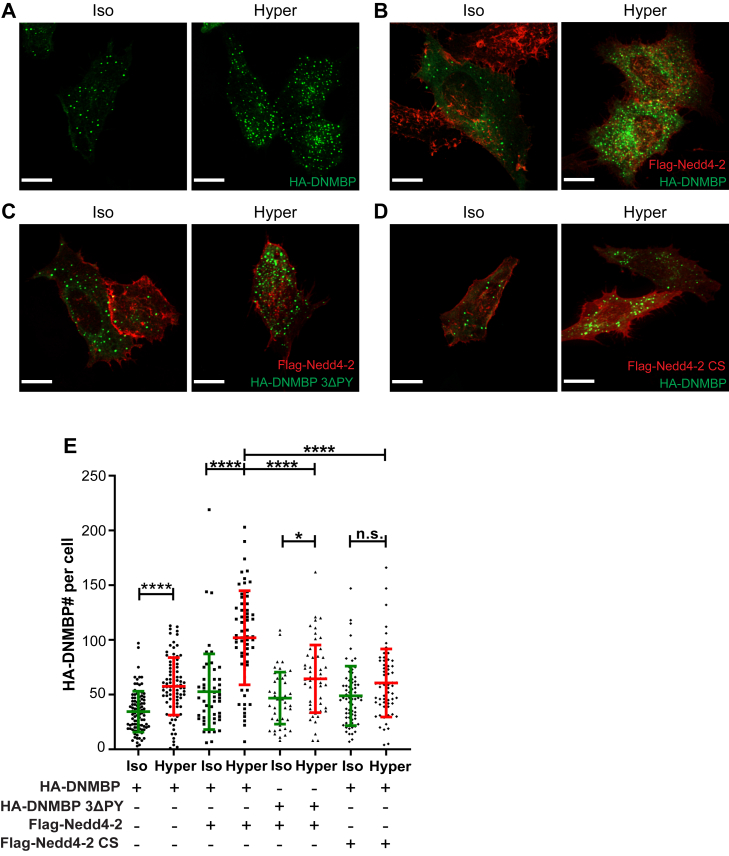
**Hyperosmolarity induces DNMBP accumulation in puncta in cells, a process that requires Nedd4-2 activity**. *A–D*, HeLa cells were transfected with HA-DNMBP (WT or 3ΔPY) and Flag-Nedd4-2 (WT or CS) for 24 h and treated with iso- or hyper-osmotic solutions for 15 min. The cells were immunostained with antibodies against HA (DNMBP, *green*) and Flag (Nedd4-2, *red*), and representative 60X confocal IF images for each condition are shown. *E*, quantification of DNMBP puncta (in *green*) per cell for each condition. *p*-values were calculated using two-way ANOVA with Tukey’s multiple comparison test. All data are mean ± sd, N = 3 independent experiments, with >50 cells quantified for each condition. *p*-values: Not significant (n.s.) > 0.05; ∗ < 0.05; ∗∗ < 0.01; ∗∗∗ < 0.001; ∗∗∗∗ < 0.0001. All scale bars are 10 μm.

Nedd4-2 is known to ubiquitinate numerous substrates, targeting them for intracellular trafficking and sometimes to degradation (19). We first investigated whether Nedd4-2 regulates the stability of DNMBP under hyperosmolarity. We found that compared to the isosmotic control, the stability of DNMBP showed a small but statistically significant decrease only after 4 h of cycloheximide (CHX) treatment under hyperosmotic conditions (Fig. S4). Given that Nedd4-2 only mildly affected DNMBP stability, which required prolonged hyperosmotic treatment, we tested whether Nedd4-2 affects DNMBP subcellular localization by performing immunofluorescence (IF) analysis using HeLa cells. Since available antibodies to endogenous DNMBP only recognize the protein in immunoblots but not in IF analyses, we expressed tagged DNMBP in cells. When expressed alone, DNMBP accumulated in puncta, but the number of DNMBP puncta was significantly greater after 15 min of hyperosmotic shock compared to the isosmotic control (Fig. 3, *A* and *E*). When we co-expressed wild-type (WT) Nedd4-2 and DNMBP, DNMBP puncta formation was increased under hyperosmolarity (Fig. 3, *B* and *E*). Moreover, this increased number of DNMBP puncta was reduced in cells expressing the catalytically inactive Nedd4-2(CS) mutant (Fig. 3, *D* and *E*). In support, cells expressing DNMBP containing the triple PY motif mutant ((3ΔPY), which poorly binds Nedd4-2) also exhibited a significant decrease in puncta formation, even when WT Nedd4-2 is expressed (Fig. 3, *C* and *E*). These findings suggest that both the binding to and ubiquitination of DNMBP by Nedd4-2 regulate DNMBP accumulation in puncta in cells exposed to hyperosmotic treatment.

### DNMBP puncta formation is enhanced by p38-MAPK and inhibited by WNK kinases

**Figure 4:**
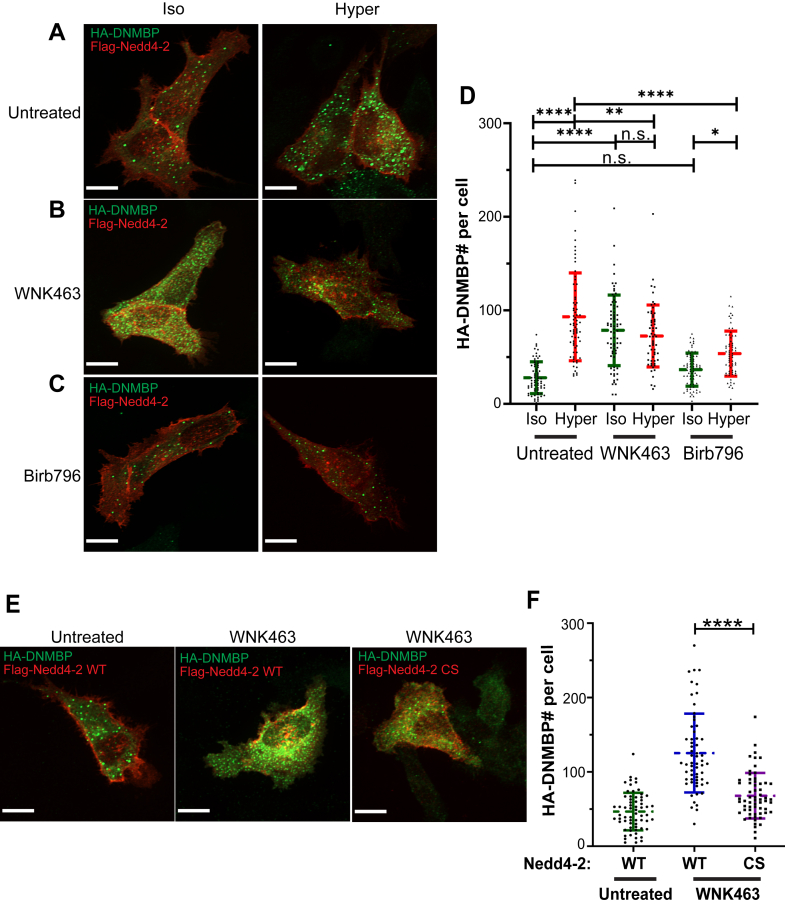
**DNMBP puncta formation is enhanced by p38-MAPK and inhibited by WNK kinases**. *A-C*, HeLa cells were transfected with HA-DNMBP and Flag-Nedd4-2 for 24 h and then treated (or not) with 10 μM WNK463 (WNK inhibitor) or 2 μM Birb796 (p38-MAPK inhibitor) for 2 h. Representative 60X confocal IF images for isosmotic and hyperosmotic conditions are shown. *D*, quantification of DNMBP puncta (in *green*) per cell after WNKs or p38-MAPK inhibition. *p*-values were calculated using two-way ANOVA with Tukey’s multiple comparison test. *E*, HeLa cells were transfected with HA-DNMBP and Flag-Nedd4-2 (WT or CS) for 24 h and then treated (or not) with 10 μM WNK463 (WNK inhibitor) for 2 h under isosmotic conditions. *F*, Quantification of DNMBP puncta (in *green*) per cell expressing Nedd4-2 (WT or CS) after WNKs inhibition with WNK463. *p*-values were calculated using an unpaired two-tailed Student’s *t* test. All data are mean ± sd, N = 3 independent experiments, with >50 cells quantified for each condition. *p*-values: Not significant (n.s.) > 0.05; ∗ < 0.05; ∗∗ < 0.01; ∗∗∗ < 0.001; ∗∗∗∗ < 0.0001. All scale bars are 10 μm.

Our previous work demonstrated that Nedd4-2 activity is enhanced by p38-MAPK and inhibited by WNK kinases (10). To determine whether these kinases regulate Nedd4-2 activity, which in turn controls DNMBP puncta formation, we treated HeLa cells with either Birb796 (p38-MAPK inhibitor) or WNK463 (pan-WNK-kinase inhibitor). Treating cells with WNK463 even under isosmotic condition led to a significant increase in DNMBP puncta formation (Fig. 4, *A*, *B*, and *D*). This increase is likely caused by elevated Nedd4-2 activity due to WNK463 inhibition. Conversely, treatment with Birb796, which was shown to reduce Nedd4-2 activity (10), resulted in decreased DNMBP puncta formation under hyperosmotic conditions (Fig. 4, *A*, *C*, and *D*). These results suggest that p38-MAPK promotes Nedd4-2 activity, thus inducing DNMBP puncta formation, whereas WNK kinases suppress Nedd4-2 activity thereby inhibiting puncta formation.

Furthermore, since WNK463 treatment may reduce cell volume and thus promote condensate formation on its own, we tested whether DNMBP puncta accumulation was dependent on Nedd4-2 activity. Thus, we expressed either Nedd4-2 (WT) or its catalytically inactive CS mutant and tested DNMBP puncta number in cells following WNK463 treatment under isosmotic conditions. Our results show that, unlike Nedd4-2(WT), the Nedd4-2(CS) mutant failed to stimulate DNMBP puncta formation (Fig. 4, *E* and *F*). This suggests that DNMBP accumulation in puncta is dependent on Nedd4-2 activity.

### DNMBP puncta do not localize to endocytic vesicles or the trans Golgi network

**Figure 5:**
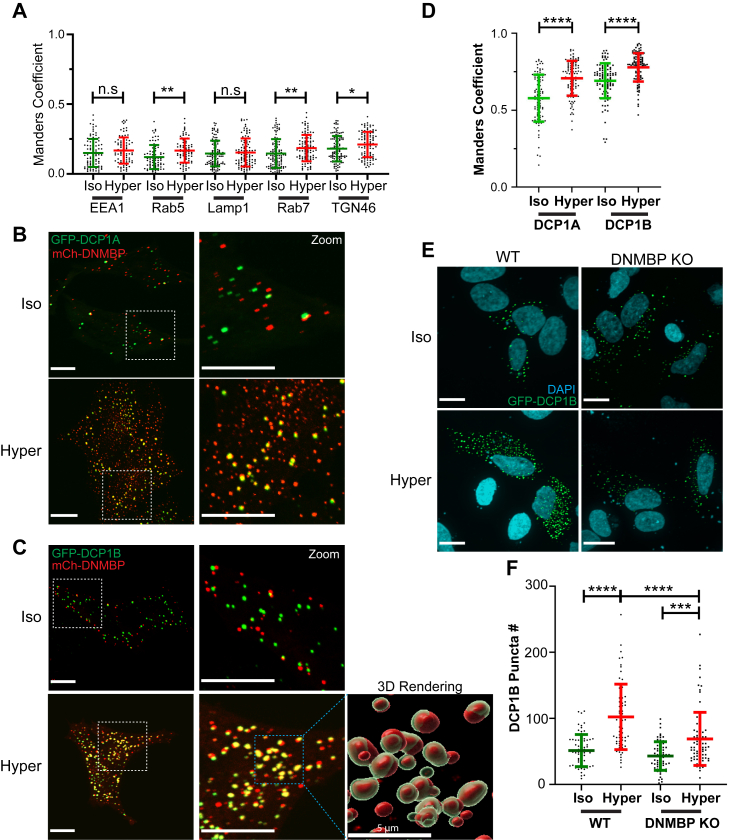
**DNMBP puncta colocalize with Processing bodies (P-bodies) markers DCP1A and DCP1B under hyperosmotic stress**. *A*, Manders colocalization coefficient was used to determine the proportion of cellular trafficking markers that colocalize with DNMBP puncta. Representative images are shown in Fig. S5*A*. *B* and *C*, HeLa cells were transfected with mCherry-DNMBP (*red*) and GFP-DCP1A or GFP-DCP1B (*green*) for 24 h and treated with iso- or hyper-osmotic cellular medium for 15 min. Representative 60X Confocal images for each condition are shown above. Scale bars are 10 μm. 3D rendering image of DNMBP (*red*) and DCP1B (*green*) colocalization was generated using Imaris software, with scale bar = 5 μm. *D*, quantification using Manders Colocalization Coefficient shows the fraction of DCP1 puncta (in *green*) that colocalizes with DNMBP puncta (in *red*) per cell. *p*-values were calculated using unpaired two-tailed Student’s *t* test. *E*, WT or DNMBP KO HeLa cells were transfected with GFP-DCP1B overnight and then treated with iso- or hyper-osmotic solutions for 15 min. Representative 60X confocal IF images for each condition are shown. Scale bars are 10 μm. *F*, quantification of DCP1B puncta (in *green*) per cell for each condition. *p*-values were calculated using two-way ANOVA with Tukey’s multiple comparison test. All data are mean ± sd, N = 3 independent experiments, >60 cells quantified for each condition. *p*-values: Not significant (n.s.) > 0.05; ∗ < 0.05; ∗∗ < 0.01; ∗∗∗ < 0.001; ∗∗∗∗ < 0.0001.

To decipher the identity of the DNMBP-containing puncta, we performed colocalization studies using antibodies targeting various intracellular compartments, including the Trans Golgi Network (TGN46), early endosomes (EEA1 and Rab5) and late endosomes/lysosomes (Rab7 and LAMP1). Using Manders Correlation to quantify co-localization between the DNMBP and the trafficking markers, we did not observe any strong colocalization (Manders Coefficient < 0.3) between DNMBP puncta and TGN46, EEA1, Rab5, Rab7, or LAMP1 in HeLa cells after hyperosmotic treatment (Figs. 5*A* an S5*A*).

### DNMBP localizes to P-bodies, but not stress granules, upon hyperosmotic treatment

Previous studies showed that hyperosmotic stress induces phase separation and leads to stress granule formation (20, 21). Therefore, we immunostained with the stress granule marker G3BP1 in cells expressing DNMBP, but did not observe any co-localization between DNMBP and G3BP1 under hyperosmolarity (Fig. S5B). Notably, recent studies have demonstrated that proteins may undergo phase separation into Processing bodies (P-bodies) after exposure to hyperosmotic stress (21). DCP1A and DCP1B are RNA decapping proteins localized to P-bodies, and have been demonstrated to undergo phase separation after hyperosmotic shock (22). Therefore, we expressed P-body-markers, GFP-tagged DCP1A or DCP1B, in HeLa cells and assessed their localization in comparison to DNMBP puncta (Fig. 5, *B* and *C*). Our work revealed a strong increase in colocalization between DNMBP and DCP1A or DCP1B after hyperosmotic treatment (Fig. 5*D*). This suggests that DNMBP likely undergoes phase separation into P-bodies in cells exposed to hyperosmotic stress.

### Depleting DNMBP in cells leads to reduced P-body formation under hyperosmolarity

Since DNMBP localizes to P-bodies under hyperosmotic treatment, we tested whether DNMBP is required for P-body formation by expressing GFP-tagged DCP1B and quantifying its numbers in both wild-type (WT) and DNMBP knockout (KO) HeLa cells. Our results show a significant increase in P-body numbers in WT cells under hyperosmotic stress (Fig. 5, *E* and *F*), consistent with previous reports (22). However, this increase was blunted in DNMBP KO cells (Fig. 5, *E* and *F*). These results suggest that DNMBP may play a role in inducing P-body formation under hyperosmolarity.

### p38-MAPK activation is reduced under hyperosmotic stress in DNMBP KO cells

**Figure 6:**
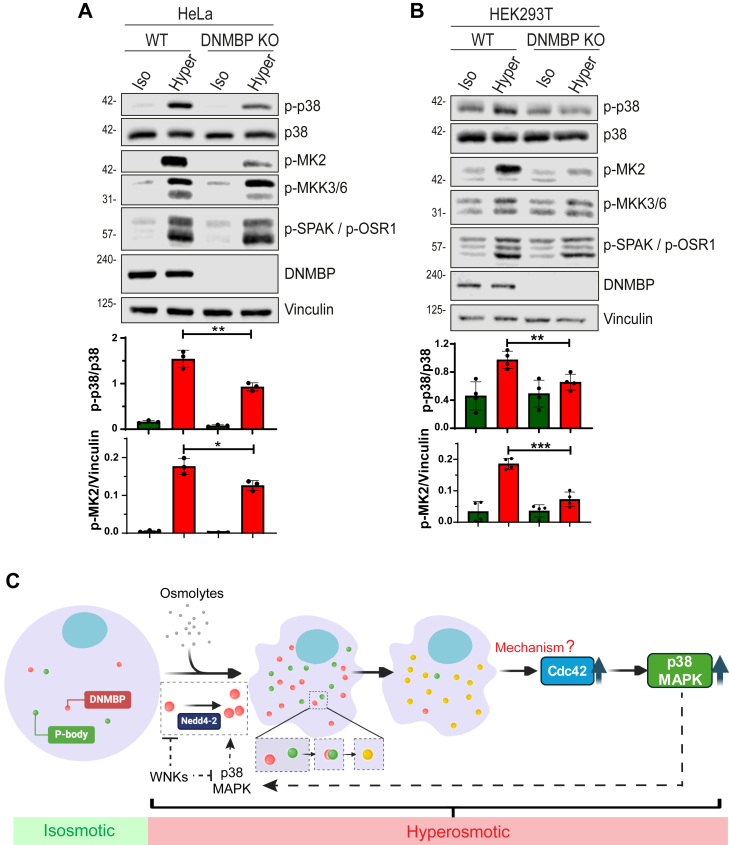
**DNMBP depletion reduces p38-MAPK activation under hyperosmotic conditions, and a Model summarizing findings of the current study**. Wild-type (WT) or DNMBP KO (*A*) HeLa cells, and (*B*) HEK293T cells, were treated with iso- or hyper-osmotic solutions for 15 min. Quantification of active (phosphorylated) p38-MAPK (p-p38) or MK2 (p-MK2) relative to their respective total proteins and loading control (vinulin) is shown below their respective blots. *p*-values were calculated using unpaired two-tailed Student’s *t* test. All data are presented as mean ± sd, N = 3 independent experiments. *p*-values: Not significant (n.s.) > 0.05; ∗ < 0.05; ∗∗ < 0.01; ∗∗∗ < 0.001. *C*, a model illustrating that Nedd4-2 induces the formation of DNMBP puncta and their localization to P-bodies under hyperosmotic stress. This process activates Cdc42, which in turn leads to the activation of the p38-MAPK pathway. Additionally, under hyperosmolarity, Nedd4-2 activity is inhibited by WNK kinases and induced by p38-MAPK. (Model was generated using BioRender).

p38-MAPK is a well-known osmotic-sensing kinase, and previous work showed that Cdc42 induces p38-MAPK activation under hyperosmotic stress (18). We investigated whether depleting DNMBP (the activator of Cdc42) affects the p38-MAPK signaling pathway by immunostaining for the active (phosphorylated) form of p38-MAPK. Our results show that in both HeLa and HEK293T cells, in contrast to WT cells, DNMBP KO leads to reduced phosphorylation of p38-MAPK (p-p38) and its downstream substrate, MK2 (p-MK2), under hyperosmotic stress (Fig. 6, *A* and *B*). Overall, these results indicate that DNMBP induces the activation of the p38-MAPK pathway (but not WNK) in a hyperosmotic environment.

## Discussion

Environmental stresses, especially hyperosmotic stress, are known to initiate cell signaling cascades that enable cells to survive and adapt to such abnormal conditions. Our previous research showed that Nedd4-2’s activity was increased following hyperosmotic treatment (10). To understand the consequences of Nedd4-2 activation under hyperosmolarity, we utilized a proximity labeling method by attaching miniTurbo to Nedd4-2 and identified its cellular interactome *via* mass spectrometry. This led to the identification of DNMBP as a substrate of Nedd4-2 during hyperosmotic stress, with the 3PY motifs on DNMBP facilitating the interaction with Nedd4-2. Additionally, we show here that the activity of Nedd4-2 (which is induced by p38-MAPK and inhibited by WNK kinases) is necessary for DNMBP to form puncta and localize to the P-body under hyperosmotic stress. Finally, we established that the activation of Cdc42 under hyperosmotic conditions is dependent on both DNMBP and Nedd4-2 (Fig. 6*C*). Interestingly, knockout of DNMBP leads to a decrease in p38-MAPK activation, which is potentially due to the reduced activity of Cdc42.

Studies from multiple groups have demonstrated that hyperosmolarity induces the formation of biomolecular condensates (22, 23). Among these, P-bodies and stress granules have been the most extensively studied (21, 24). Phosphorylation is the most commonly studied post-translational modification that directs proteins to these membrane-less compartments (21). However, the connection between ubiquitination and biomolecular condensates has been less extensively studied. Currently, the most prominent role of ubiquitination is in the disassembly of P-bodies and stress granules. Previous work indicated that the ubiquitination of G3BP1, an essential component of stress granules, leads to the destabilization of stress granules (25). Regarding the initiation of biomolecular condensate formation, the K63 linkage of ubiquitination has been demonstrated to contribute to the formation of P-bodies. Point mutation on K63 residues on endogenous ubiquitin results in reduced P-body numbers (26). Importantly, Nedd4 proteins are known to ubiquitinate their substrates with a K63 linkage (27). In this study, we show that wild-type Nedd4-2 induces DNMBP localization to puncta, whereas the catalytically inactive mutant (CS) does not. Although colocalization between Nedd4-2 and DNMBP was not observed in our IF experiments, our Co-IP results demonstrated that Nedd4-2 interacts with and ubiquitinates DNMBP. We therefore propose that Nedd4-2 ubiquitinates DNMBP in the cytosol, thus targeting it to P-bodies under hyperosmotic stress. This is consistent with the previous research showing that K63 ubiquitination is crucial for targeting DCP1A to the P-body (26).

DCP1A is a critical component of the mRNA decapping complex (21). It was proposed earlier that under stress conditions, mRNAs are concentrated and sequestered within the P-bodies, thereby permitting the initiation of mRNA decay (21, 26). Mutations in DCP1A have been demonstrated to decrease P-body formation and reduce decapping activity (26). Another potential role of P-bodies is shielding misfolded or active proteins from proteasome degradation. This protection requires DCP2, a major component of the mRNA decapping complex (28, 29). Interestingly, when we knocked out DNMBP in cells, the number of P-bodies was also reduced. This implies that DNMBP might be an important element in P-bodies formation, but its exact role in P-body remains unclear.

Earlier research demonstrated that the small Rho GTPases, Rac1 and Cdc42, become activated under hyperosmolarity (3, 18). However, the GEF(s) responsible for activating Rac1 and Cdc42 under these conditions has/have remained unclear. In this study, we show that DNMBP and Nedd4-2 are essential components for the activation of Cdc42 under hyperosmotic stress, as depletion of either Nedd4-2 or DNMBP leads to a reduction in Cdc42 activity. We hypothesize that Nedd4-2 ubiquitinates DNMBP in the cytosol, targeting it to the P-body, which in turn induces Cdc42 activation (Fig. 6*C*). However, we were unable to confirm the colocalization between DNMBP and Cdc42 in cells because of the diffuse staining pattern of the ectopically expressed Cdc42 observed under confocal microscopy, precluding proper quantification of colocalization.

Additionally, we discovered that p38-MAPK activation was downregulated upon knockout of DNMBP under hyperosmolarity, suggesting that removing DNMBP reduces Cdc42 activation, consequently reducing activation of the p38-MAPK pathway. This finding aligns with a previous study, which demonstrated that p38-MAPK is activated by Rac1 and Cdc42 under hyperosmotic stress (18). Here, our result confirms that Cdc42 functions as an upstream regulator of the p38-MAPK pathway. Moreover, the activation of Cdc42 is dependent on Nedd4-2 and its ubiquitination on DNMBP.

The fact that p38-MAPK promotes puncta formation (likely by enhancing Nedd4-2 activity) and this, in turn, promotes p38-MAPK activation under hyperosmotic stress (Fig. 6*C*), is puzzling, as it is likely to create a feed-forward cycle of p38-MAPK activation. What then stops this cycle? We suspect that either WNK (itself activated by hyperosmolarity), which we showed earlier to inhibit p38-MAPK and Nedd4-2 activity (10, 30), and/or restoration of iso-osmolarity *via* regulatory volume increase (RVI) after hyperosmotic shock, could stop this feed-forward cycle.

Taken together, our work here has identified substrates of Nedd4-2 in cells under hyperosmotic stress, with DNMBP being an important one. We then showed that Nedd4-2 ubiquitinates DNMBP and targets it to P-bodies under hyperosmolarity. This localization may play an important role in activating Cdc42 and its downstream p38-MAPK pathway.

## Experimental procedures

All reagents used in this study are listed in the experimental procedures (Table S1).

### Cell culture

HeLa, HEK293T, and 293 Flp-In T-REx cells were cultured in Dulbecco's Modified Eagle Medium (DMEM) supplemented with 10% fetal bovine serum (FBS) and antibiotic-antimycotic solution. 293 Flp-In T-REx stable cell lines were maintained in media containing 100 μg/ml hygromycin B. Transfections were performed using PolyJet reagents. For isosmotic *versus* hyperosmotic treatment, cells were treated with either normal cellular medium (DMEM + 10% FBS) with an osmolarity of 330 mOSM, or hyperosmotic cellular medium (DMEM + 10% FBS + 135 mM NaCl) with an osmolarity of 600 mOSM. All cell lines used in this study were tested and confirmed to be free of *mycoplasma*.

### Immunoblotting, Co-immunoprecipitation (Co-IP) assays, and ubiquitination assays

HEK293T or HeLa cells were transfected with the specified cDNA constructs for 2 days prior to treatment. Subsequently, cells were treated with either isosmotic or hyperosmotic cellular medium for 15 min and lysed in lysis buffer (50 mM Hepes, pH 7.5, 150 mM NaCl, 1% Triton X-100, 10% glycerol, 1.5 mM MgCl_2_, 1.0 mM EGTA, and 10 μg/ml of each leupeptin, aprotinin, and pepstatin, and 1 mM PMSF). Co-IP experiments were performed by immunoprecipitating 1 mg of cleared cell lysate using the specified antibody conjugated with affinity beads. Proteins were separated on SDS-PAGE and immunoblotted with the indicated antibodies. For the ubiquitination assay, cell lysates were boiled for 5 min in 1% SDS to remove any proteins in complex with HA-DNMBP. HA-DNMBP was then immunoprecipitated using an anti-HA antibody conjugated with affinity beads and probed for ubiquitin. All immunoblots were imaged using the Odyssey Imaging system (Odssey Fc, LI-COR) and quantified using Image Studio version 5.2 (LI-COR). Co-IP experiments were quantified by normalizing the Co-IP protein to the IP protein and then to the expression level of the Co-IP protein in the cell lysate. Phosphorylated proteins were normalized to their corresponding total protein levels.

### DNMBP PY motif mutagenesis

The pcDNA3-HA-DNMBP construct (Table S1) was used to create point mutations in the PY motifs of DNMBP. These mutants were generated through site-directed mutagenesis and verified by sequencing. All recombinant DNA constructs used in this study are listed in Table S1.

### Generation of Nedd4-2 knockdown (KD) HEK293T cells

HEK293T cells were transfected with a human Nedd4-2 shRNA construct (Table S1). The cells were then selected with Puromycin (2 μg/ml) 24 h after transfection. Cellular medium (containing Puromycin) was changed every 2 to 3 days. Once the stably transfected cells were established, single colonies were picked out and expanded. Nedd4-2 knockdown was verified by immunoblotting with Nedd4-2 antibodies.

### Generation of DNMBP CRIPSR knockout (KO) HeLa and HEK293T cells

HeLa or HEK293T cells were transfected with a PX459 plasmid containing Cas9, Puromycin resistance gene and a gRNA targeting DNMBP (Table S1). Transfected cells were selected with puromycin (2 μg/ml) for 48 h. Then, single colonies were picked and expanded in 96-well plates with cell culture medium lacking puromycin. After expansion, DNMBP knockout was confirmed by immunoblotting with DNMBP antibodies. Clones g1-5 and g2-5 were used for all experiments as they showed a complete knockout of DNMBP in immunoblots.

### Cdc42 activation assay (Cdc42 G-lisa kit)

Cdc42 activation was measured and quantified using the Cdc42 G-lisa kit (Cytoskeleton, Inc). Briefly, wild-type (WT), Nedd4-2 knockdown (KD), or DNMBP knockout (KO) HEK293T cells were treated with isosmotic or hyperosmotic cellular medium for 15 min and then lysed using the lysis buffer provided in the kit. The cell lysates were then centrifuged, the supernatants collected and flash frozen. Upon collecting all replicates, the samples were thawed in a room-temperature water bath and loaded onto a 96-well plate containing the Cdc42-GTP-binding protein. The active form of Cdc42 (bound to GTP) adhered to the plate, whereas the inactive form (bound to GDP) was removed through washes. Subsequently, the bound active Cdc42 was detected using a Cdc42-specific antibody.

### Immunofluorescence (IF) sample preparation and microscopy

HeLa cells were seeded on 12-well plates containing glass coverslips. The cells were transfected with the indicated constructs overnight and then treated with either isosmotic or hyperosmotic cellular medium for 15 min. For the kinase inhibition experiments, the cells were treated with either 10 μM WNK463 (pan-WNK-kinase inhibitor) or 2 μM Birb796 (p38-MAPK inhibitor) for 2 h prior to hyperosmotic treatments. Cells were then fixed with 4% paraformaldehyde in PBS for 15 min, permeabilized with 0.1% Triton-X for 10 min, blocked with 5% milk and then stained with the indicated primary antibody, fluorophore-conjugated secondary antibody and DAPI. The samples were mounted on a cover glass using DAKO mounting medium. A IX81 confocal microscope (Olympus) with a 60X/1.35 oil immersion or 40X/0.95 water immersion objective was used to acquire the Z-stacked IF images. Images were processed and quantified using Volocity (version 7.0.0, Quorum Technologies). The 3D rendering image was produced using Imaris (version 10.1.1, Oxford Instruments).

### BioID (miniTurbo) sample preparation

Tetracycline-inducible, miniTurbo-tagged Nedd4-2 were stably expressed in 293 Flp-In T-REx cells using the Flp-In site-specific recombination system. To create the stable cell lines, Nedd4-2 was first cloned into the pcDNA5-FRT-TO plasmid, tagging Flag-miniTurbo at either the N-terminus (FmT-Nedd4-2) or the C-terminus (Nedd4-2-mTF). 293 Flp-In T-REx cells were then co-transfected with pOG44 (Flp-recombinase expression vector) and the pcDNA5-FRT-TO plasmid, which contains Nedd4-2 fused with Flag-miniTurbo. Stable cell lines were generated by selection with 200 μg/ml hygromycin B. The expression of fusion proteins in the resulting isogenic cell pools was validated by immunoblotting. Two independent pools (biological replicates) were created for each condition. Stable isogenic cell pools expressing FmT-Nedd4-2 or Nedd4-2-mTF were expanded to five 15 cm^2^ plates. The expression of the fusion protein was induced by the addition of 1 μg/ml tetracycline to the culture media for 24 h. Cells were then treated with iso- or hyper-osmotic solutions with 50 μM biotin for 1 h and were subsequently collected by scraping and centrifugation. Cell pellets were washed twice with PBS and stored at −80 °C until lysis. Biotin-streptavidin affinity purification was performed as previously described (31).

### Mass spectrometry

High-performance liquid chromatography was performed using a 2 cm pre-column (Acclaim PepMap; 50 mm × 100 μm inner diameter (ID)) and a 50 cm analytical column (Acclaim PepMap; 500 mm × 75 μm ID; C18; 2 μm; 100 Å; Thermo Fisher Scientific, Waltham, MA). The system ran a 120-min reversed-phase buffer gradient at 225 nl/min on a Proxeon EASY-nLC 1000 pump, interfaced with a Thermo Q-Exactive HF quadrupole-Orbitrap mass spectrometer. Parent ion scans were conducted at a resolving power of 60,000, after which up to the twenty most intense peaks were selected for MS/MS (minimum ion count of 1000 for activation) using higher energy collision-induced dissociation (HCD) fragmentation. Dynamic exclusion was applied such that MS/MS of the same *m/z* (within a range of 10 ppm; exclusion list size = 500) detected twice within 5 s were excluded from analysis for 15 s. For protein identification, Thermo.RAW files were converted to the.mzXML format using Proteowizard (32) and searched using X!Tandem (33) and COMET (34) against the Human RefSeq Version 45 database (containing 36,113 entries). Data analysis was performed using the trans-proteomic pipeline (TPP) (35) *via* the ProHits software suite (v3.3) (36). Search parameters included a parent ion mass tolerance of 10 ppm and an MS/MS fragment ion tolerance of 0.4 Da, allowing up to two missed cleavages for trypsin. Variable modifications of +16@M and W, +32@M and W, +42@N-terminus, and +1@N and Q were included. Proteins identified with an iProphet cut-off of 0.9 (corresponding to ≤1% FDR) and at least two unique peptides were analyzed with SAINT Express v.3.6 (37). Twenty control runs (from cells treated with isosmotic condition) were condensed to the four highest spectral counts for each prey and compared to the experimental data, consisting of two biological replicates (each analyzed with two technical replicates). High-confidence interactors were defined as those with a Bayesian false discovery rate (BFDR) ≤0.01. All raw mass spectrometry data files have been deposited at the MassIVE archive (massive.ucsd.edu) accession MSV000097232.

### Quantification and statistical analysis

Immunoblots were visualized using the Odyssey Imaging system and quantified with Image Studio version 5.2 (LI-COR). Manders Colocalization Coefficient and puncta counting in immunofluorescence experiments were conducted using Volocity (version 7.0.0, Quorum Technologies). Each experiment was repeated at least three times independently. Statistical analysis was performed using GraphPad Prism 8. Histograms show mean ± sd; *p*-values < 0.05 were considered statistically significant and are indicated in the Figures (*p* values: Not significant (n.s) > 0.05, ∗ < 0.05, ∗∗ < 0.01, ∗∗∗ < 0.001, ∗∗∗∗ < 0.0001, using unpaired Student t-tests or ANOVA tests, as indicated in the figure legends).

## Data availability

All data supporting the findings of this study are available from the corresponding author upon request. Raw mass spectrometry data files for the BioID experiment have been deposited at the MassIVE archive (massive.ucsd.edu) accession MSV000097232.

## Supporting information

This article contains supporting information.

## Conflict of interest

The authors declare that they have no conflict of interest with the contents of this article.
