# Supplementary material for: The ubiquitin ligase Nedd4-2 promotes localization of DNMBP/Tuba to P-bodies under hyperosmotic stress
Source: J Biol Chem. 2025 Sep 18;301(11):110738. doi: 10.1016/j.jbc.2025.110738 (PMC12569825; doi:10.1016/j.jbc.2025.110738)
Supplement: Supplemental data [file mmc1.docx]

**SUPPORTING INFORMATION**

**The ubiquitin ligase Nedd4-2 promotes localization of DNMBP/Tuba to P-bodies under hyperosmotic stress**

Zetao Liu^1,2^, Chong Jiang^1^, Faith Yeung^3,4^, Brian Raught^3,4^ and Daniela Rotin^1,2,*^

^1^Cell & Systems Biology Program, The Hospital for Sick Children, Toronto, Ontario, Canada

^2^Department of Biochemistry, University of Toronto, Toronto, Ontario, Canada

^3^Princess Margaret Cancer Centre, University Health Network, University of Toronto, Toronto, Ontario, Canada

^4^Department of Medical Biophysics, University of Toronto, University of Toronto, Toronto, Ontario, Canada

*To whom correspondence should be addressed:

Dr. Daniela Rotin, The Hospital for Sick Children, 686 Bay St., Toronto, Ontario, Canada M5G 0A4

Email: drotin@sickkids.ca

Tel: (416) 813-5098

**SUPPLEMENTARY TABLES**

**Table S1. Key resources of chemical, reagents and antibodies**

| REAGENT or RESOURCE | SOURCE | IDENTIFIER |
| --- | --- | --- |
| Antibodies | | |
| Mouse anti-DNMBP | Abcam | Cat# ab88534 |
| Mouse anti-β-actin | Sigma-Aldrich | Cat# A2228 |
| Mouse anti-V5 | Bio-Rad | Cat# MCA1360G |
| Mouse anti-V5 | Thermo-Fisher | Cat# R96025 |
| Rabbit anti-V5 | Cell Signaling | Cat#13202s |
| Mouse anti-HA | Biolegend | Cat# 901515 |
| Rabbit anti-HA | Cell Signaling | Cat# 3724S |
| Mouse anti-ubiquitin | Santa Cruz | Cat# SC-8017 |
| Rabbit anti-Nedd4-2 | Cell Signaling | Cat# 4013S |
| Mouse anti-phospho-p38 MAPK (T180/Y182) | BD Bioscience | Cat# 612280; RRID:AB_399597 |
| Mouse anti-vinculin | Santa Cruz | Cat# sc-25336; RRID:AB_628438 |
| Rabbit anti-phospho-SPAK antibody (Ser-373)/phospho-OSR1 antibody (Ser-325) | EMD Millipore | Cat# 07-2273 |
| Mouse anti-SPAK (STK39) | EMD Millipore | Cat# MABS178 |
| Rabbit anti-phospho-MKK3 (Ser189)/MKK6 (Ser207) | Cell Signaling | Cat# 12280 |
| Rabbit anti-p38 MAPK | Cell Signaling | Cat# 9212; AB_330713 |
| Rabbit anti-phospho-MAPKAPK-2 (p-MK2) (Thr334) | Cell Signaling | Cat# 3007 |
| Mouse anti-FLAG | Sigma | Cat# F1804-5MG |
| Rabbit anti-DYKDDDDK (FLAG) | Cell Signaling | Cat# 14793S |
| Rabbit anti-EEA1 | Cell Signaling | Cat# 3288T |
| Mouse anti-Rab5A | Cell Signaling | Cat# 46449T |
| Rabbit anti-Lamp1 | Cell Signaling | Cat# 9091T |
| Rabbit anti-Rab7 | Cell Signaling | Cat# 9367S |
| Sheep anti-TGN46 | MyBioSource | Cat# MBS1750080 |
| Mouse anti-G3BP1 | BD Transduction Labs | Cat# 611126 |
| Alexa Fluor 488 anti-mouse | Thermo Fisher Scientific | Cat# A11001 |
| Alexa Fluor 488 anti-rabbit | Thermo Fisher Scientific | Cat# A11008 |
| Alexa Fluor 594 anti-mouse | Thermo Fisher Scientific | Cat# A11005 |
| Alexa Fluor 594 anti-rabbit | Thermo Fisher Scientific | Cat# A11037 |
|  |  |  |
| Chemicals, Peptides, Transfection reagents and Recombinant Proteins | | |
| Birb796 | SelleckChem | Cat# S1574 |
| WNK463 | SelleckChem | Cat# S8358 |
| Puromycin | Bioshop | Cat# PUR333 |
| Hygromycin B | Invitrogen | Cat# 10687010 |
| Tetracycline | BioShop | Cat# TET701 |
| Biotin | BioShop | Cat# BIO302 |
| DAPI | Roche | Cat# 10236276001 |
| DAKO Fluorescence Mounting Medium | DAKO | Cat# S3023 |
| DMEM | Wisent | Cat# 319-005-CL |
| Fetal bovine serum | Wisent | Cat# 098450 |
| Antibiotic-antimycotic | Wisent | Cat# 450-115-EL |
| PolyJet In Vitro DNA Transfection Reagent | SignaGen Laboratories | Cat# SL100688 |
| BLUelf Prestained Protein Ladder | FroggoBio | Cat# PM008-0500 |
| Anti-FLAG M2 Affinity gel | Sigma | Cat# A2220-5ML |
| QuikChange Site-Directed Mutagenesis Kit | Agilent | Cat# 200518 |
| Cdc42 G-lisa Kit | Cytoskeleton, Inc. | Cat# BK127 |
| Rhodamine Phalloidin | Invitrogen | Cat# R415 |
|  |  |  |
| Plasmids and Constructs | | |
| HA-DNMBP | Addgene | Cat# 22214 |
| HA-DNMBP (Y145A) | ‘This study’ |  |
| HA-DNMBP (Y479A) | ‘This study’ |  |
| HA-DNMBP (Y1269A) | ‘This study’ |  |
| HA-DNMBP (3ΔPY) | ‘This study’ |  |
| mCherry-DNMBP | Addgene | Cat# 129622 |
| Flag-Nedd4-2 WT | (Persaud et al, 2022, PMID: 35858421) |  |
| Flag-Nedd4-2 CS |  |  |
| V5-Nedd4-2 CS |  |  |
| V5-Nedd4-2 CS |  |  |
| GFP-DCP1A | Addgene | Cat# 153972 |
| GFP-DCP1B | Addgene | Cat# 153976 |
| LAMTOR1-Flag | Addgene | Cat# 42331 |
| Flag-LAMTOR2 | Addgene | Cat# 42330 |
| Flag-ARHGAP1 | Human ORFeome V5.1 Gateway entry clones,  provided by SPARC Drug Discovery | Acc# BC018118 |
| Flag-BAIAP2 (I3) |  | Acc# BC032559 |
| Flag-BAIAP2 (I6) |  | Acc# BC014020 |
| Flag-BAIAP2L1 |  | Acc# BC013888 |
| Flag-CXADR |  | Acc# BC003684 |
| Flag-EPS8 |  | Acc# BC030010 |
| Flag-STX6 |  | Acc# BC009944 |
| Flag-STX10 |  | Acc# BC017237 |
| Flag-TMOD1 |  | Acc# BC002660 |
| Flag-TMOD2 |  | Acc# BC064961 |
| Flag-TMOD3 |  | Acc# BC020542 |
| Experimental Models: Cell Lines | | |
| HeLa | ATCC | Cat#CCL-2 |
| HEK293T | ATCC | Cat#CRL-1573 |
| 293 Flp-In T-REx | Thermo Fisher Scientific | Ca# R78007 |
| DNMBP CRISPR KO gRNA sequence | | |
| g1: caccATCACCCTTACGGTACTTG |  |  |
| g2: caccAGGTGACTGCTAACTCGGT |  |  |
| g3: caccTTGACCGCAAGGACTGCAT |  |  |
| N-ter: caccAGGTTATAAAACATGGAGGC |  |  |
| C-ter1: caccAGTACACCTGAGCCCACGTT |  |  |
| C-ter2: caccCAAAACCGAGTACACCTGAG |  |  |
|  |  |  |
| Nedd4-2 shRNA | | |
| GIPZ Lentiviral Human NEDD4L shRNA | Horizon | V3LHS_300779 (ORF) |
| GIPZ Lentiviral Human NEDD4L shRNA | Horizon | V2LHS_80459 (ORF) |
| GIPZ Lentiviral Human NEDD4L shRNA | Horizon | V2LHS_80461 (ORF) |
|  |  |  |
| Software and Algorithms | | |
| Syngistix | PerkinElmer |  |
| Prism 8 | GraphPad |  |
| Image StudioVersion 5.2 | Li-Cor |  |
| Volocity 7.0.0 | Quorum Technologies |  |
| Zen | Zeiss |  |
| Imaris Image Analysis Software | Oxford Instruments |  |

**SUPPLEMENTARY FIGURES:**

**
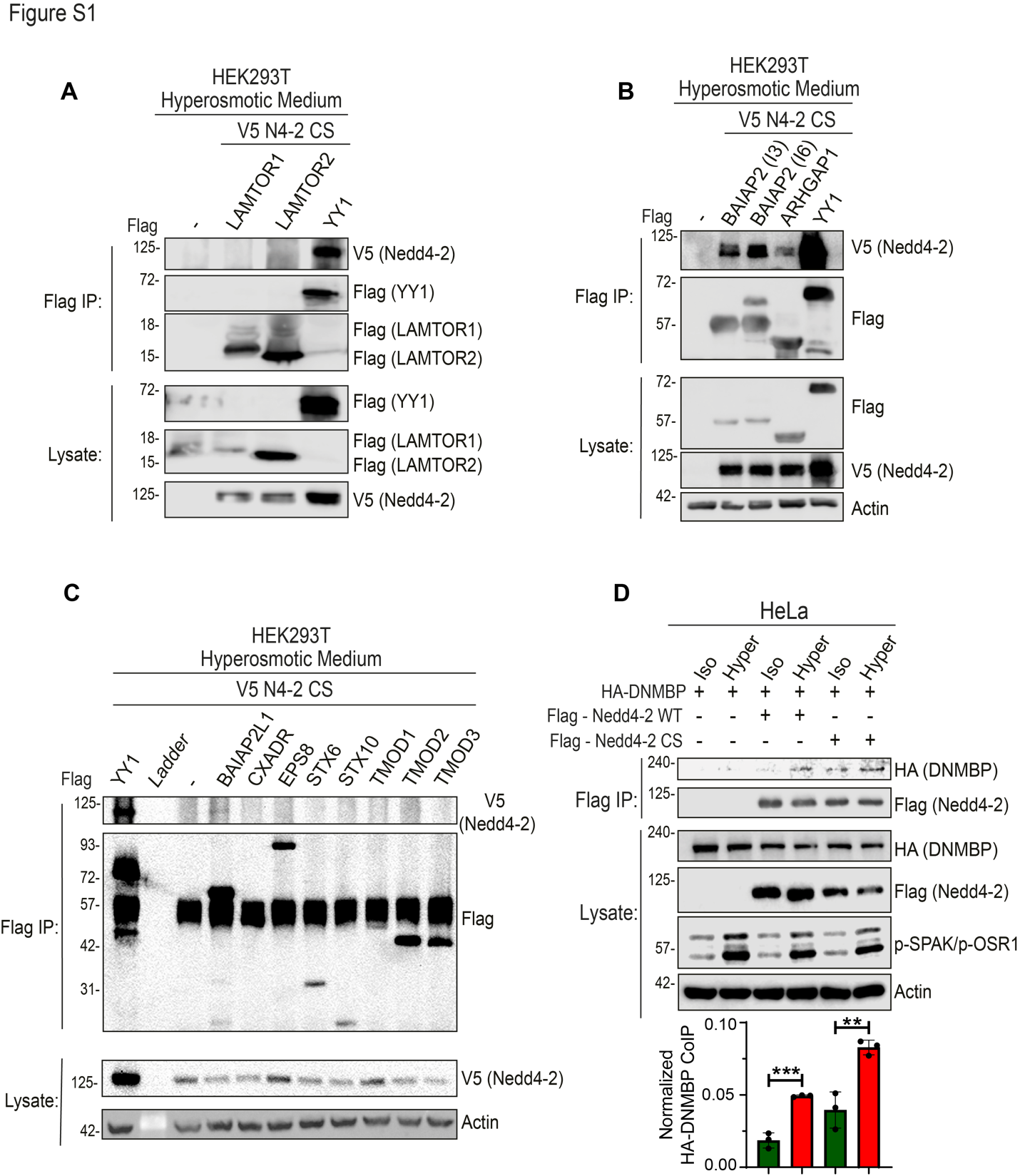
**

**Supplementary Figure 1. Validation of Nedd4-2 interactions with several top-hit proteins from the BioID/miniTurbo screen.**

HEK293T cells were transfected with V5 tagged Nedd4-2 and/or Flag tagged **(A)** LAMTOR1 or LAMTOR2; **(B)** BAIAP2 (isoform 3) or BAIAP2 (isoform 6); **(C)** BAIAP2L1, CXADR, EPS8, STX6, STX10, TMOD1, TMOD2 or TMOD3, for 48 hrs and treated with hyperosmotic solution for 15 min. Top-hit proteins were immunoprecipitated (IP) using affinity beads conjugated with anti-Flag antibody. Co-immunoprecipitation (Co-IP) of Nedd4-2 was validated by immunoblotting for V5. YY1 was used as the positive control. Note that the BAIAP2 isoforms were not further studied because their binding to Nedd4-2 was not affected by osmolarity changes. Blots are representatives of 2-3 separate experiments. (**D**) HeLa cells were transfected (or not) with HA-tagged DNMBP and Flag-tagged Nedd4-2 constructs (WT or the catalytically-inactive CS mutant) for 48 hrs and treated with iso- or hyper-osmotic cellular medium for 15 min. Nedd4-2 was immunoprecipitated (IP) with affinity beads conjugated with anti-Flag antibodies, and HA-DNMBP Co-immunoprecipitation (Co-IP) was determined by immunoblotting for HA. p-values were calculated using unpaired two-tailed Student’s t-test. Quantification is depicted below the respective blots. All data are mean±sd, N=3 independent experiments. p-values: Not significant (n.s.) > 0.05; * < 0.05; ** < 0.01; *** < 0.001.


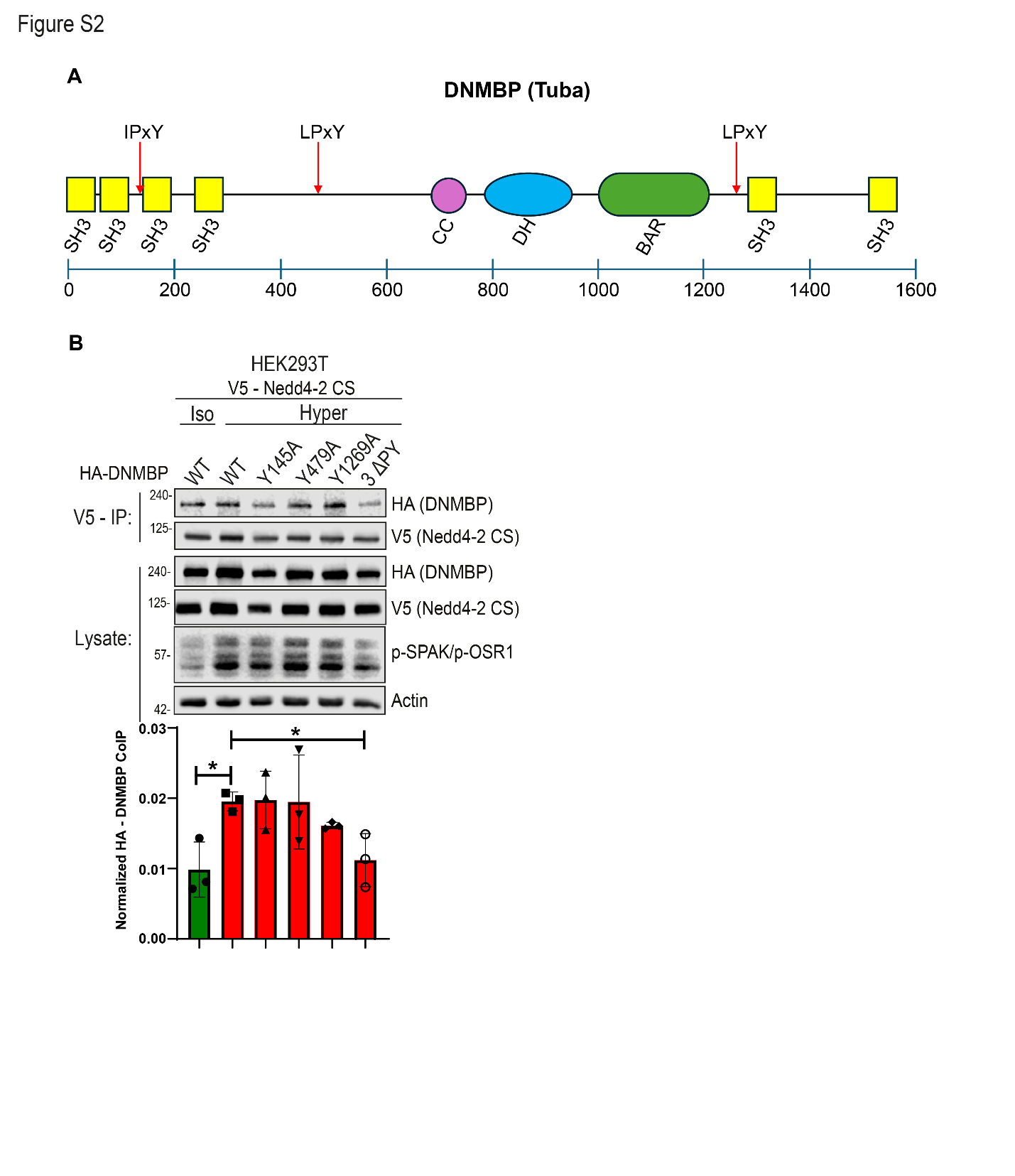


**Supplementary Figure 2. Nedd4-2 interacts with DNMBP via its 3 PY motifs**

**(A)** Schematic illustration of DNMBP domains, its 3 PY motifs and its other domains. **(B)** HEK293T cells were transfected with V5-Nedd4-2 and HA-DNMBP (WT, Y145A, Y479A, Y1269A, and 3ΔPY) and then treated with iso- or hyperosmotic solutions for 15 min. V5-Nedd4-2 was immunoprecipitated with V5-conjugated beads, and HA-DNMBP Co-immunoprecipitation was determined by immunoblotting. p-values were calculated using one-way ANOVA test. All data are presented as mean ± sd, N=3 independent experiments. p-values: Not significant (n.s.) > 0.05; * < 0.05.


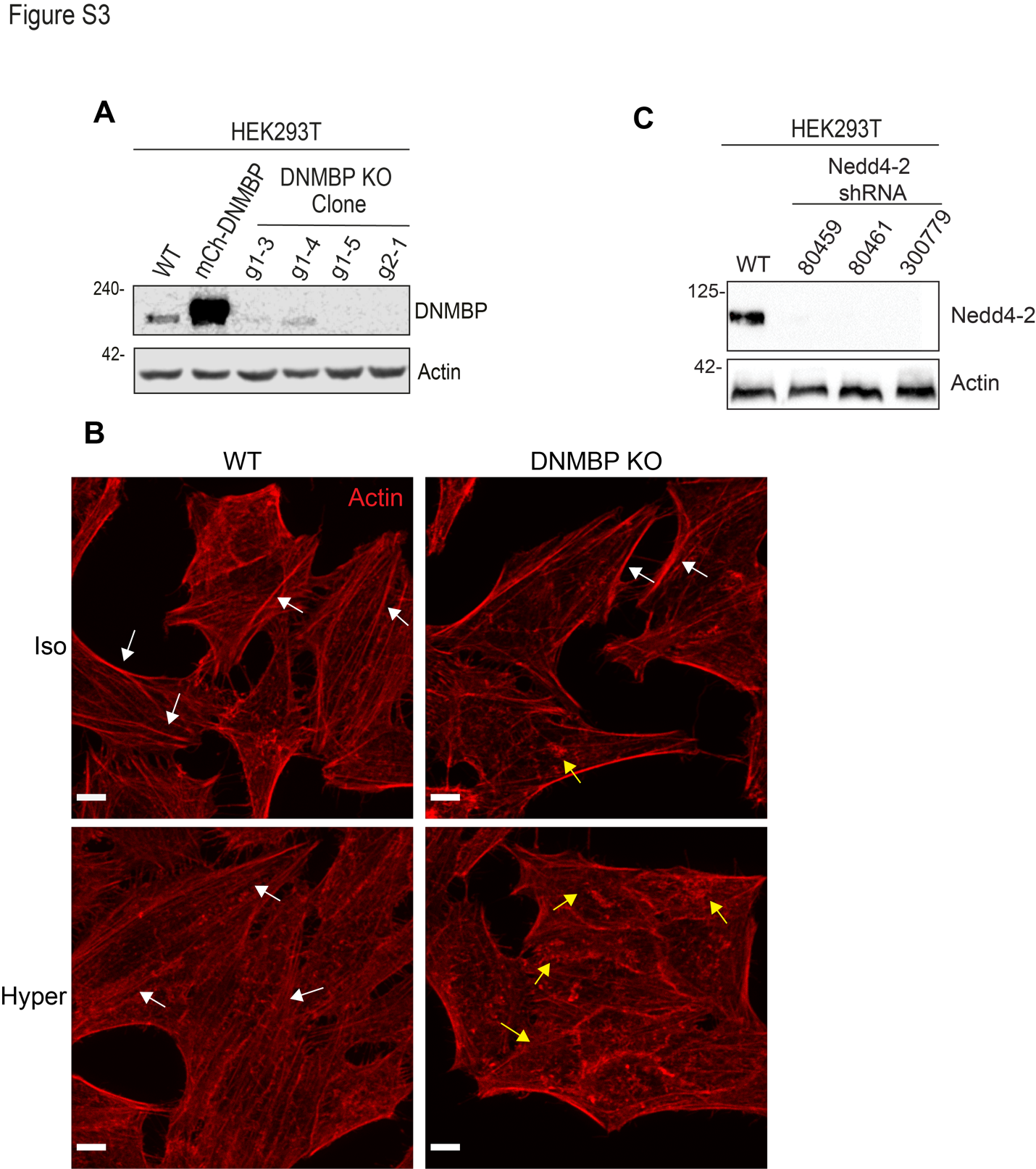


**Supplementary Figure 3.** **Generation of** **DNMBP knockout (KO) and Nedd4-2 knockdown (KD) HEK293T cells, and actin filaments disruption in the DNMBP KO cells.**

**(A)** DNMBP knockout (KO) HEK293T stable cell lines were generated using CRISPR-Cas knockout. Clones g1-5 and g2-5 were used for all experiments as they showed a complete knockout. **(B)** WT or DNMBP KO HeLa cells were treated with iso- or hyper-osmotic medium for

15 minutes, followed by phalloidin staining to visualize actin (red). White arrows indicate filamentous actin, and yellow arrows indicate disrupted or punctate actin structures. Representative 40X confocal IF images are shown for each condition. N = 3 independent experiments, with 45 fields imaged per condition. All scale bars are 10 μm**. (C)** Nedd4-2 knockdown (KD) HEK293T stable cell lines were generated through shRNA transfection, followed by puromycin selection.


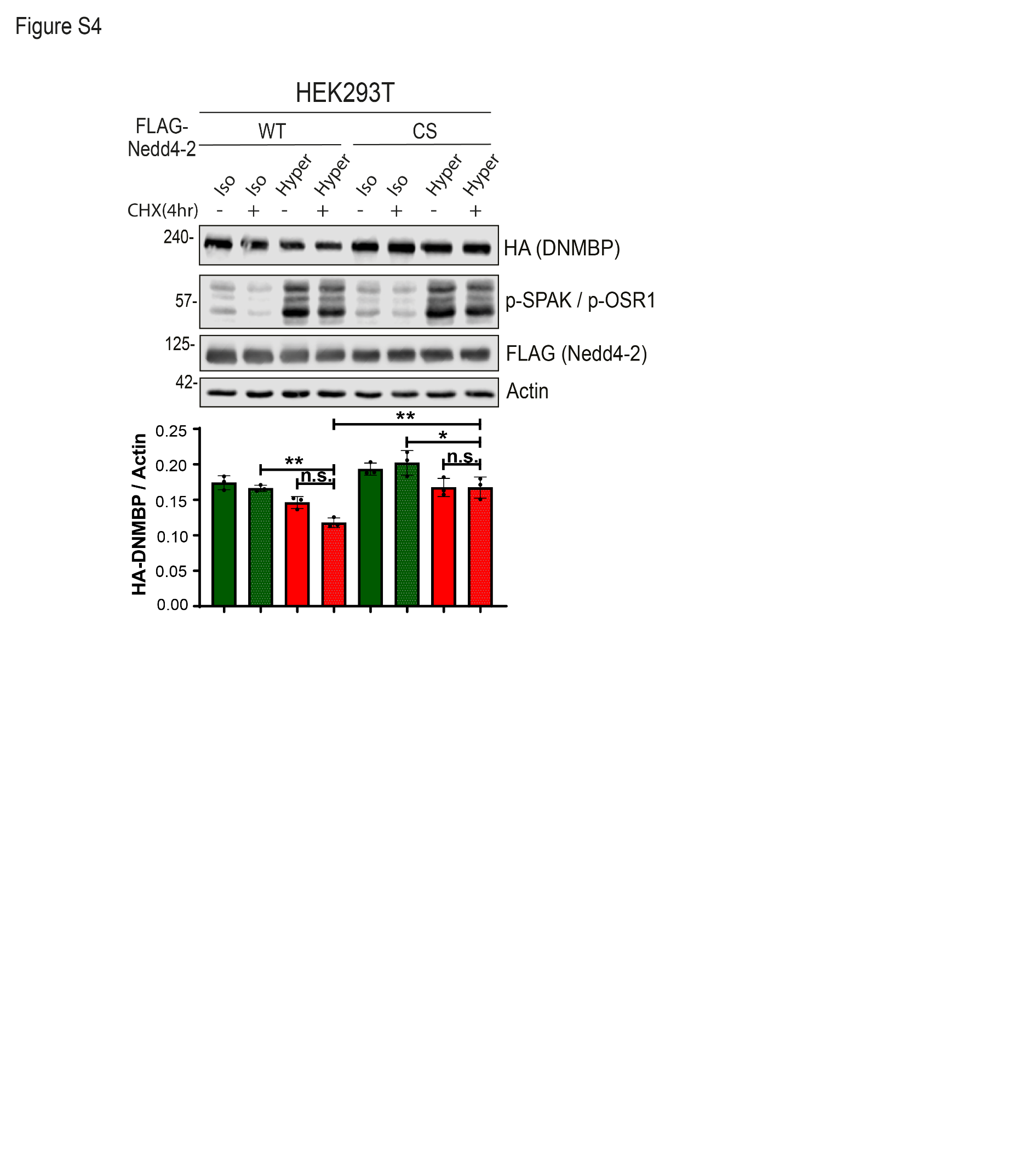


**Supplementary Figure 4. Regulation of DNMBP stability by Nedd4-2.**

HEK293T cells were transfected with HA-DNMBP or Flag-Nedd4-2 (WT or CS) for 48 hrs and then treated (or not) with cycloheximide (CHX) for 4 hrs. Cells were then treated with iso- or hyper-osmotic solutions for 15 min. HA-DNMBP protein level was quantified by normalizing to the loading control (actin). p-values were calculated using two-way ANOVA test with Tukey’s multiple comparison test. All data are presented as mean ± sd, N = 3 independent experiments. p-values: Not significant (n.s.) > 0.05; * < 0.05; ** < 0.01.


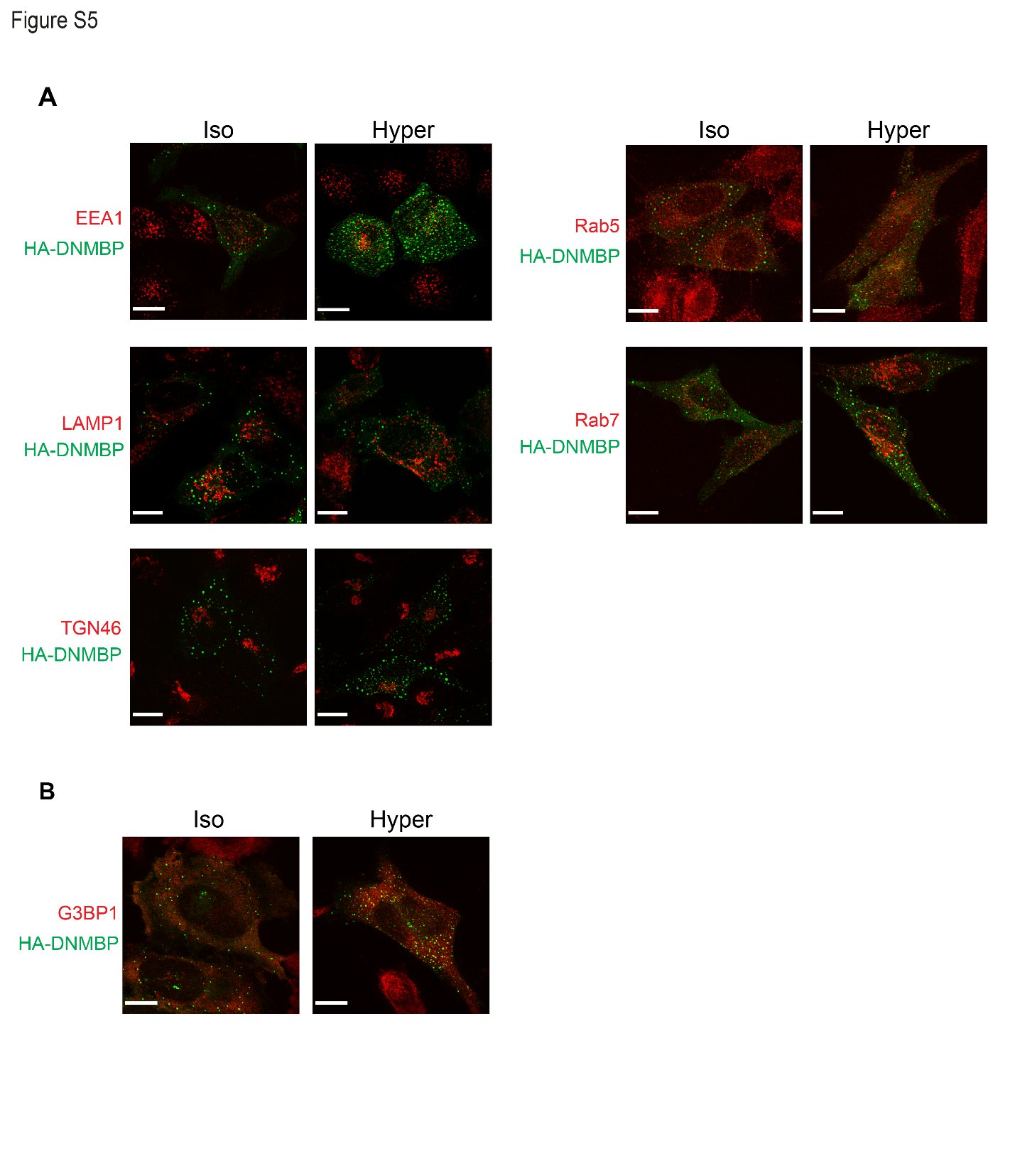


**Supplementary Figure 5. DNMBP does not colocalize with cellular trafficking and stress granules markers.**

**(A)** HeLa cells were transfected with HA-DNMBP (green) and treated with iso- or hyper-osmotic solutions for 15 min. Cellular trafficking markers were stained with the indicated antibodies (EEA1, Rab5, LAMP1, Rab7, and TGN46, red). The Manders Coefficient between DNMBP and the trafficking markers was quantified and is shown in Figure 5A. **(B)** HeLa expressing HA-DNMBP and treated with iso or hyper-osmotic solutions for 15 min. Stress granules were stained with antibodies to G3BP1. Scale bars are 10 μm.
